# Microstructure and Properties of Self-Assembly Graphene Microcapsules: Effect of the pH Value

**DOI:** 10.3390/nano9040587

**Published:** 2019-04-10

**Authors:** Yan-Dong Guo, Jun-Feng Su, Ru Mu, Xin-Yu Wang, Xiao-Long Zhang, Xin-Ming Xie, Ying-Yuan Wang, Yi-Qiu Tan

**Affiliations:** 1 Department of Polymer Material, School of Material Science and Engineering, Tianjin Polytechnic University, Tianjin 300387, China; YandongGuoTJPU@163.com (Y.-D.G.); zhangxiaolong2019@163.com (X.-L.Z.); xiexinming2019@163.com (X.-M.X.); 2 School of Civil and Transportation Engineering, Hebei University of Technology, Tianjin 300401, China; muru2019@163.com; 3 School of Mechanical Engineering, Tianjin University of Commerce, Tianjin 300134, China; xinyuwang2019@163.com; 4 School of Transportation Science and Engineering, Harbin Institute of Technology, Harbin 150090, China; wangyingyuan2019@163.com (Y.-Y.W.); tanyiqiu2000@163.com (Y.-Q.T.)

**Keywords:** graphene, microcapsule, self-assembly, pH value, microstructure

## Abstract

Graphene has attracted attention in the material field of functional microcapsules because of its excellent characteristics. The content and state of graphene in shells are critical for the properties of microcapsules, which are greatly affected by the charge adsorption equilibrium. The aim of this work was to investigate the effect of pH value on the microstructure and properties of self-assembly graphene microcapsules in regard to chemical engineering. Microcapsule samples were prepared containing liquid paraffin by a self-assembly polymerization method with graphene/organic hybrid shells. The morphology, average size and shell thickness parameters were investigated for five microcapsule samples fabricated under pH values of 3, 4, 5, 6 and 7. The existence and state of graphene in dry microcapsule samples were analyzed by using methods of scanning electron microscope (SEM), transmission electron microscope (TEM) and X-ray photoelectron spectroscopy (XPS). Fourier Transform Infrared Spectoscopy (FT-IR) and Energy Dispersive Spectrometer (EDS) were applied to analyze the graphene content in shells. These results proved that graphene had existed in shells and the pH values greatly influenced the graphene deposition on shells. It was found that the microcapsule sample fabricated under pH = 5 experienced the largest graphene deposited on shells with the help of macromolecules entanglement and electrostatic adherence. This microcapsules sample had enhanced thermal stability and larger thermal conductivity because of additional graphene in shells. Nanoindentation tests showed this sample had the capability of deforming resistance under pressure coming from the composite structure of graphene/polymer structure. Moreover, more graphene decreased the penetrability of core material out of microcapsule shells.

## 1. Introduction

Functional microcapsules have drawn considerable attention in scientific and industrial research [1]. Active compounds, inorganic material or organic material can be microencapsulated using chemical, physicochemical, or mechanical methods [2]. These core-shell structure particles exhibit significant physical changes in response to environmental stimuli such as temperature [3], pH [4,5,6,7], light irradiation [8], electric [9], magnetic fields [10], redox [11] and mechanical stress [12]. Microcapsules are tiny core-shell particles with large surface area and inner volume, which can develop environment-responsive sensitive and rapid stimuli in response. This intelligent feature provides a lot of functional material characteristics. Therefore, microcapsules have stimulated great interest for researchers in recent years in many fields, such as thermal energy storage [13], chemical separation [14], food protection [15], drug delivery [16], biosensors [17], self-healing materials [18], self-lubricating materials [19], anti-corrosion coating [20], anti-icing coating [21], fire resistive materials [22], battery materials [23], superabsorbent materials [24], CO_2_ capture [25] and so on.

The research contents of microcapsules can be divided into two aspects: fabrication of novel functional microcapsules and characterization properties of functional microcapsules [26]. For the first aspect, many works have been carried out to microencapsulate new and particularly core materials or to develop novel methods of microencapsulation. Normal chemical microencapsulation methods include in-situ polymerization, interface polymerization and emulsion polymerization [27]. An increasing interest has been ignited in areas of supramolecule encapsulation [28] and self-assembly [29]. It is critical to prove that the microcapsules have appropriate functional properties using correct test methods. Accordingly, the selection of the encapsulation procedure will greatly depend on the average particle size, the core agent, the shell material and the cost of the microcapsule product. It is important to understand the relationship between the capabilities and properties, because the application of microcapsules greatly depends on their different properties. Furthermore, nearly all properties of microcapsules are determined by the shell microstructure. For example, phase change materials were microencapsulated and used for absorbing-releasing thermal energy in the thermal energy storage field [2]. It is desired for microcapsules to retain a compact structure without rupturing, which leads to a suffering thermal expansion of the core material and temperature change. The thermal stability and mechanical property of shells is pivotal. In the self-healing, self-lubricating and anti-corrosion systems, microcapsules may release the active ingredient via external mechanical force or chemical force. The mechanical properties of the shell are crucial for these functional microcapsules. Other than the controlled release system, nearly all microcapsules are possess a tight and compact shell structure. It can be determined that the structure parameters of microcapsules including shell thickness, average size and microstructure all affect their mechanical strength. Several techniques are available for enhancing the shell mechanical strength and compactness, such as cross-linkage degree improvement [30], polymer blend [31], and metal or inorganic particle additive [32]. Because each characterization method of physicochemical and structural for microcapsules has its own limitations, the information and accuracy requirement will determine the choice of characterization technology. It needs to be mentioned that the inorganic nano-particle addictive, such as nano-CaCO_3_ and nano-SiO_2_, is an ideal approach to dramatically improve the thermal stability, mechanical property and compactness of microcapsules [33]. 

Publications and patents of graphene research are explosively growing and arousing worldwide interest due to graphene’s peculiar physical characteristics. Graphene has a unique and peculiar structure and properties, which are different from other well-known materials. It is the thinnest two-dimensional (2D) carbon material with a single layer of carbon atoms and a specific surface area of 2600 m^2^·g^−1^ [34]. Also, it has the highest thermal conductivity of 3000 W·m^−1^·K^−1^ and the largest conductivity of 6 × 10^5^ S·m^−1^. Furthermore, graphene possesses excellent mechanical properties, such as a 130 GPa tensile strength and a 1.0 TPa Young’s modulus [35]. Due to the aforementioned excellent characteristics of graphene, this field has attracted a large number of material researchers to devote a great deal of effort towards the application of graphene. Graphene is now also a popular nanomaterial in fields of electronics, energy and biology. Interestingly, it is also being considered as an ideal application for improving the characters of functional microcapsules [36]. Initially, graphene oxide was applied to form microcapsules with inorganic/organic shells, which were applied in the fields of self-healing material [37], thermal energy storage [38], drug delivery [39] and photoresponsive release [40]. Functional groups of organic shell materials can have a chemical connection with graphene oxide, forming a stable composite structure. Graphitic oxide can also serve as easily suspended and processed inorganic particles in aqueous media. However, it has lower thermal conductivity than graphene. In addition, the non-electroconductivity of graphene oxide also limits its application scope of microcapsules. Therefore, graphene endows microcapsules with more functional properties, simulating research interest. Su [2] successfully prepared microcapsules containing a core material with a solid-liquid phase change transfer ability by a self-assembly polymerization with graphene shells. A graphene/organic hybrid shell was formed based on the process of electric charge absorption and long-molecular entanglement. The properties of microcapsules had dramatically improved because of the existence of graphene. For example, microcapsules possess a sensitivity response to temperature due to the excellent thermal conductivity of graphene. Phase-change temperature of microcapsules was regulated because graphene reduced the thermal barrier of the polymer shells. The Young module of shells had also been enhanced due to the addition of graphene. Recently, another graphene/organic nano-hybrid microcapsule product was reported as providing electrothermal self-healing for aged bitumen [41]. These microcapsules had an excellent electric conductivity capability because of the advent of graphene in shells. It has been further reported that epoxy resin/phosphorus-based microcapsules were fabricated through an in-situ polymerization as a flame retardation product with high-density polyethylene/graphene shells [9].

Although graphene has been considered as an additive to enhance the physical function of microcapsule product, it is still hard for it to deposit on microcapsule shells because its monolayer carbon atom structure does not have any chemical reaction with the organic molecule compared to graphene oxide. In-situ polymerization (O/W, oil/water) is a normal preparation method for microcapsules with graphene or graphene oxide [2,9]. In detail, core material is dispersed by a violent stirring into small droplets with micrometer or nanometer size. The droplets in water ultimately form a stable emulsion with the help of amphiphilic emulsifier. For example, hydrolyzed (SMA) molecules were successfully applied to emulsify core materials including paraffin [2] and other oily liquid [9]. Then the surface of droplets was gathered with positive charges. As these macromolecules had negative functional groups, electrostatic interaction forces the macromolecules to be adsorbed on the droplet surfaces [9]. Interestingly, electrostatic attraction could also lead the inorganic nano-particles to adhere on the droplet surface with the help of chain entanglement [33]. Under a certain pH value and temperature, macromolecules were cross-linked by acid, forming nano-inorganic/organic shells at an equilibrium point. In previous work [2,9], it has been proved that the graphene depositing on shells is based on the joint actions of electrostatic interaction and molecule entanglements. Initially, graphene sheets have been entangled by macromolecules in a hydrolyzed state. It is worth noting that the graphene deposition should be happening with a maximum amount under a certain pH value [9]. In other words, the acid-based equilibrium determines the deposition amount of graphene on shells. In the case of microencapsulated phase change material, the shell may need as much graphene as possible in shells. More graphene definitely accelerates the thermal transmission speed [2]. As for thermalelectrical microcapsules, enough graphene is needed in shells to form an electrically conductive path [41]. In other cases, the graphene amount should be an appropriate amount to regulate the shell properties such as mechanical character, penetration capability and thermal stability. At the same time, the pH value in emulsion greatly affects the hydrophilic-lipophilic balance (HLB) of emulsifier. In another hand, the HLB value of an emulsifier is a measure of the degree of hydrophilic or lipophilic determined by calculating values for different regions of the molecule. The graphene deposition on droplets is greatly influenced at the same time by the HLB values. Hence, it is a key issue to control the graphene content in shells by regulating the acid-base equilibrium.

Inspired by the broad application prospects of graphene microcapsules, the aim of this work was to prepared microcapsules with self-assembly graphene in shells. In view of chemical engineering, the effect of the pH values of polymerization in emulsion was systematically investigated on the microstructure and characteristics of microcapsule samples. The detail of self-assembly polymerization process was observed under pH values between of 3 to 7. Microstructure and properties of microcapsule samples were significantly affected by the pH values. In this work, the physicochemical microstructure of microcapsules was investigated to ensure the graphene content was adequate. The thermal stability and thermal conductivity of microcapsules were measured. The mechanical property of single microcapsule was tested by nanoindentation technology. The compactability of microcapsules was evaluated by a penetration method. Properties information is helpful to understand the graphene self-assembly process of influencing by pH values. Furthermore, these results can be used to guide the production of graphene functional microcapsules in chemical engineering.

## 2. Experimental

### 2.1. Materials

Graphene was a commercial product supplied by Tuling Co., Ltd. (Shenzhen, China). Paraffin was used as the core material (0.905 g/cm^3^, 4.24 Pa·s, Tianjin Sinogo. Co., Ltd., Tianjin, China). Prepolymer hexamethoxymethylmelamine (HMMM) was used as the polymeric shell material and was supplied by Tianjin Sinogo. Co., Ltd. (solid content of 98.0%, China). A copolymer of styrene maleic anhydride (SMA) was purchased as a dispersant (Hercules, New York, NY, USA). Other chemicals are chemically pure reagents.

### 2.2. Preparation of Microcapsules

The graphene microcapsule samples were fabricated by a self-assembly method with the following four steps [2,9]: (1) SMA powder was hydrated in hot water (50 °C) with a pH value of 10, which was controlled by a 5.0 wt.% NaOH solution. Oily paraffin was added into the surfactant solution and dispersed into droplets under a vigorous stirring for 10 min. (2) HMMM prepolymer and graphene were mixed homogeneously using a ultrasonic dispersion method. Then it was dropwisely added into the above mixture paraffin emulsion with a 500 r·min^−1^ stirring speed. This dropwise addition method can help to achieve a perfect shell structure of microcapsules with a relatively low reaction rate [9]. At the same time, graphene has enough time to deposit on droplets through electrical charge and HMMM molecule entanglement. The temperature of emulsion was gradually raised to 40 °C with a speed of 2 °C·min^−1^. (3) pH value of above solution was adjusted to 3–7 by a 1.0 wt.% ethylic acid dropwisely. The temperature of solution was raised from 40 °C to 80 °C with a speed of 2 °C·min^−1^. Temperature was naturally cooled to room temperature after a 3 h cross-linking polymerization of HMMM. (4) Through filtration, cleaning and drying, the microcapsule product was finally obtained. 

### 2.3. Morphology Analysis of Microcapsules

The formation details of microcapsules in emulsion was observed by a biological microscope (XSP-2CA, Sanfeng Co. Changzhou, China). The dried microcapsules were adhered on a double-side adhesive tape without cracking the shells. The surface morphologies were observed by using a scanning electron microscope (SEM, FEI Nanosem-430, Hillsboro, OR, USA) at an accelerated voltage of 20 kV. The state of graphene in shells was observed by a transmission electron microscope (TEM, Hitachi HT7700, city, Japan). 

### 2.4. Shell Thickness and Size Distribution

Laser particle size analyzer (JHY-1076, Jinheyuan, Xiamen, China) was utilized to measure the size distribution of microcapsules. In order to measure the shell thickness of shells, a gelatin composite sample was made with microcapsules (10.0 g) dispersing in it. Cross-section slides of the composite were obtained using an ultramicrotome (FC7-UC7, Leica, Solms, Germany). Shell thickness could be easily measured under a microscope. The average value of fifty thickness data was calculated as the value of the shell thickness of the certain microcapsule sample.

### 2.5. Chemical Structure of Shells

The chemical functional groups of shell material were analyzed using a FT-IR (Perkin Elmer Spectrometer 100, PerkinElmer Instrument Co., Ltd., Waltham, MA, USA) spectra in absorption modes recorded among the range of 400–4000 cm^−1^. An energy dispersive spectrometer (EDS, APOLLO XL, EDAX, Mahwah, NJ, USA) was applied to determined the elements ratios of C, N, and O in microcapsule shells. 

### 2.6. Thermal Properties of Microcapsules

The thermal stability characterization of microcapsule samples was analyzed by a thermogravimetic analysis (TGA, SDT-2960, Dupont, Wilmington, DE, USA) in a flow of 40 mL·min^−1^ nitrogen (N_2_) with a scanning rate of 5 °C·min^−1^.

The thermal conductivity of microcapsule powder was measured by a thermal conductivity tester (DRXL, Jiezhun Instrument Equipment Co., Ltd., Shanghai, China) referencing ASTM E-1530 Standard. Microcapsule powder was spread between the two polished surfaces held. A pressure was forced on the surfaces. Heat flowed from the upper surface to the lower surface through the powder sample establishing an axial temperature gradient in the stack. The temperature difference was measured based on the heat output from the heat flow transducer at a thermal equilibrium. The heat data and powder thickness were used to calculate the thermal conductivity according to Equation (1)

(1) $$\Phi =A\frac{\lambda }{h}\left(T_{1}-T_{2}\right)$$

where *Φ* is the heat, *λ* is thermal conductivity, *A* is the area of powder, *h* is the thickness of powder, and *T*_1_ and *T*_2_ are temperatures of upper surface to the lower surface. 

Thermal transmission phenomena were observed by using an infrared thermograph (IRT, HT-02, XinTest, Shenzhen, China). A microcapsule sample (5.0 g) was put on a constant temperature heating plate with temperature of 100 °C. Thermal images were taken every 5 s during 50 s. The heat conduction law was analyzed by comparing these infrared thermographs. 

### 2.7. Nanoindentation Tests

**Figure 1:**
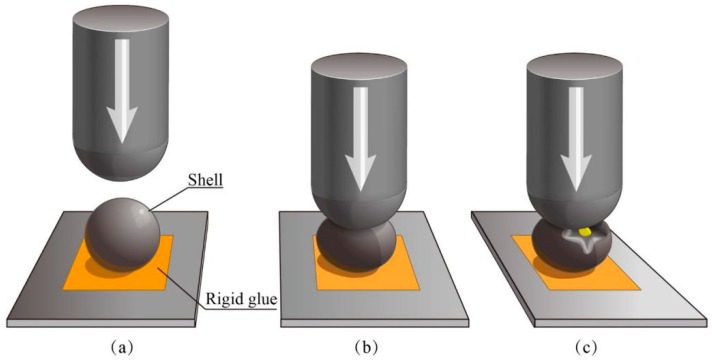
Illustration of nanoindentation method testing the mechanical property of single microcapsule, (**a**) a microcapsule rigid glued on a plate, (**b**) the elastic deformation on the microcapsule under pressure, and (**c**) a plastic deformation on the microcapsule under pressure.

A Nano Indenter^®^ XP (MTS Nano Instruments, Keysight Technologies Inc., Palo Alto, CA, USA) cone tip (3 μm radius) was out on a single microcapsule with a pressure as illustrated in Figure 1a,b. The force was set as 50 nN holding 10 s and displacement resolutions were 0.01 nm [26,41]. A load-displacement curve plotted out based on these output mechanical values with a yield stress. It has been proved that the microcapsule has a plastic deformation after its yield point (Figure 1c). The break behaviors of microcapsules were not discussed in this work.

### 2.8. Compactability of Shells

Penetration capability of paraffin out of microcapsules was evaluated using a UV visible spectrophotometer (LAMBDA 950, PerkinElmer Instrument Co., Ltd., Waltham, MA, USA). In this work, ethyl alcohol was applied as a good extraction solvent, which easily extracted the core material of paraffin. The relationship between the penetration time and the residual weight of paraffin was plotted based on the transmittance of UV light. The optical density (*P*) of the dispersing medium is measured and converted into the concentration of paraffin using a calibration curve,

(2) $$P_{R}=\frac{P_{0}-P_{t}}{P_{0}}\times 100\%$$

where *P_R_* is the residual weight of paraffin still in microcapsules, *P*_0_ is the *P* value of all encapsulated paraffin in ethyl alcohol and *P*_t_ is the *P* value of encapsulated paraffin in ethyl alcohol at a point of permeation time (*t*). A qualitative analysis was carried out to compare the resistance penetration capability of different microcapsule shells with graphene [41]. 

## 3. Results and Discussion

### 3.1. Morphologies of Microcapsules in Emulsion and Dry States

Four parameters usually are characterized for a self-assembled microcapsule product, including the average size, shell thickness, shell microstructure and shell strength [2]. These characteristics can be controlled by regulating the details of the polymerization process. For example, it was found that the surface of microcapsules was smoother under a lower addition rate of shell material. Under a lower dropping rate of shell materials, the shell strength was significantly enhanced [26]. The reason is that a lower shell material dropping rate leads to a slower polymerization ratio of shells with fewer defects. The shell thickness is primarily determined by the core/shell ratios [9]. Besides the shell structure, the average size of microcapsules can be controlled by regulating the stirring rate of emulsion [33]. The emulsion stirring rates determine the average size of microcapsules [2,9,33]. To simplify the complexity of the above parameters of microcapsules, five typical microcapsule samples were prepared at the emulsion stirring rate of 2000 r·min^−1^. The core/shell material (paraffin/HMMM) ratio is 1/2 and the graphene/HMMM ratio is 5.0% as listed in Table 1. M-0 is the microcapsule sample fabricated without graphene under a polymerization condition with pH value of 4. Others are coded as M-3, M-4, M-6 and M-7, which are fabricated under the pH value of 3, 4, 5, 6 and 7 respectively. All microcapsule samples have the graphene/HMMM ratio (wt.%) of 5.0%. It should be mentioned that not all graphene can deposit on shells. Normally, melamine-formaldehyde (MF) was widely applied to prepare various functional microcapsule products because of its low price, easy technology, high compactness, fire resistance and chemical resistance [33]. However, the residual formaldehyde in cross-linked MF resin is harmful to health and the environment. To overcome this problem, HMMM prepolymer was applied in this work to fabricate various microcapsule samples with a hybrid graphene/HMMM shells. Cross-linked HMMM resin almost completely lacks residual formaldehyde due to a sufficient chemical reaction [33].

**Figure 2:**
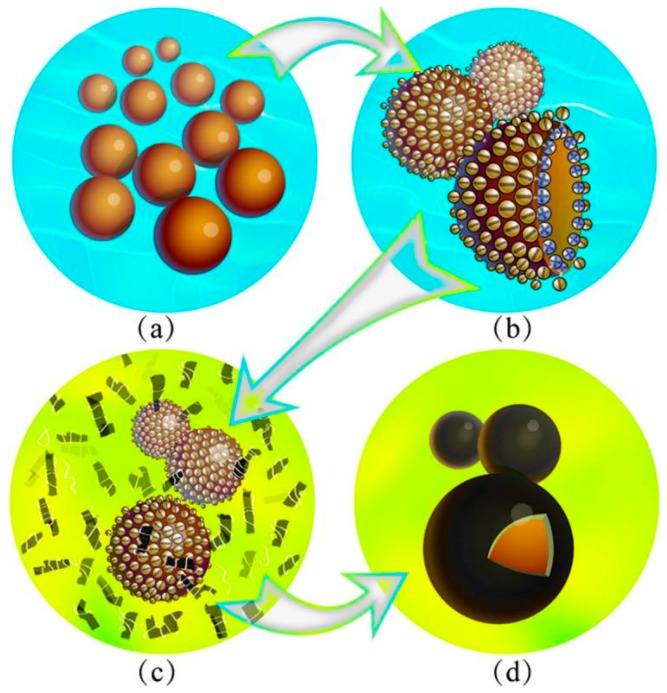
Illustration of microcapsules fabrication process by a self-assembly method, (**a**) core material emulsified by SMA molecules, (**b**) the emulsified droplets with negative charge on surface, (**c**) HMMM prepolymer and graphene adsorbed on core droplets by charge, HMMM prepolymer added dropwise and attached to the surface of the droplets, droplets with positive charge, and (**d**) three-dimensional anatomical structure of microcapsules with graphene/HMMM shells.

Figure 2 illustrates the fabrication process of microcapsules in emulsion state. SMA molecules can be hydrolyzed (pH > 7) with a negative charge. Then oil phase droplets are dispersed under a certain stirring, forming an O/W emulsion (Figure 2a). Subsequently, hydrolyzed SMA molecules are attached to the inner phases of the droplet interface. In detail, the hydrophilic carbonyl ends are extended to the aqueous phase and the lipophilic ends insert the interior of the droplets [9,33]. Paraffin droplets are ultimately dispersed by hydrolyzed SMA molecules with both of hydrophilic ends and lipophilic ends (Figure 2b). Functional groups of HMMM prepolymer dissolve in an acid aqueous solution with lots of negative charge by combining with hydrogen ion. Hence, electrostatic interaction definitely leads the HMMM molecules absorbing on the droplet surfaces (Figure 2c). At the same time, graphene is adsorbed on the surface of the droplets by both forces of chain entanglement and electrostatic attraction. Under a high temperature and the proper pH value, HMMM prepolymer molecules cross-links form a hybrid composite shell with graphene (Figure 2d). 

**Figure 3:**
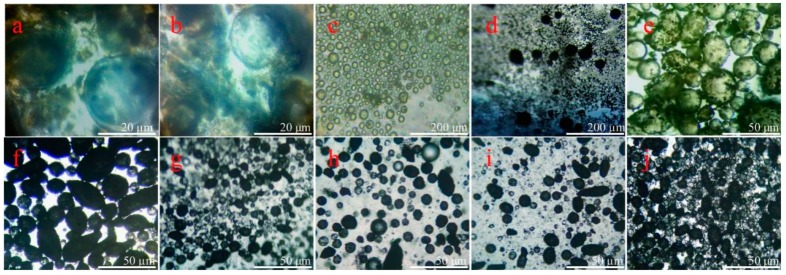
Optical microscope morphologies of core droplets in emulsion emulsified by hydrated SMA molecules, (**a**) core material dispersed into droplets by a stirring, (**b**,**c**) core droplets emulsified by SMA, (**d**,**e**) graphene and HMMA adsorbed on droplets, (**f**) microcapsules with graphene/HMMM shells; microcapsule samples in emulsion: (**g**) M-4, (**h**) M-5, (**i**) M-6, and (**j**) M-7.

Figure 3 shows the optical microscope morphologies with more details of the formation process for microcapsules. In the first step, the core material is dispersed by a stirring into droplets (Figure 3a). With the help of hydrolyzed SMA as polymeric surfactant, the droplets are emulsified forming a stable O/W solution (Figure 3b,c). Because hydrolyzed SMA molecule acts as an emulsifier in water with sufficiently amphiphilic adsorbing on liquid droplets, it definitely has greatly influenced the microstructure of shells. In particular, the amount of SMA can control the compactability of shells, and thus, leads to stable, non-leaking spherical microcapsules [32]. In the second step, the solution of HMMA molecule is dropped into the emulsion, which is adsorbed on the core material droplets by the electrostatic adsorption. At the same time, graphene sheets deposit on the surface of droplets with the help of chain entanglements (Figure 3d,e). Under an acid conation, the HMMM molecules polymerize to a cores-linked structure. Graphene is kept in the cross-linking structure, forming a hybrid graphene/HMMM composite (Figure 3f). Figure 3g–j shows the optical microscope morphologies of the microcapsule samples (M-4, M-5, M-6 and M-7) in emulsion. In view of intuitive observation, M-5 has a relatively smooth surface and a regular spherical shape. 

**Figure 4:**
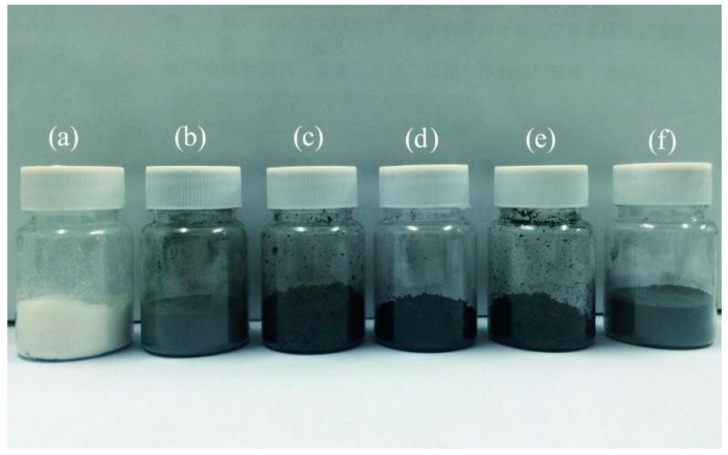
Optical images of various dry microcapsule powders: (**a**) M-0, (**b**) M-3, (**c**) M-4, (**d**) M-5, (**e**) M-6, and (**f**) M-7.

Figure 4 displays a photograph of various microcapsule samples with different colors. As the core material of paraffin is a colorless transparent liquid, the M-0 sample has a white color (Figure 4a). The black color of microcapsule samples gets deeper and deeper (M-3, M-4 and M-5) (Figure 4b–d). Because the natural color of graphene is black, it implies that more graphene may have successfully deposited on shells with the increasing of pH values of polymerization. The hybridization process and electric charge may determine this graphene deposition on shells. However, both M-6 and M-7 have a relative color shift (Figure 4e,f). It may be inferred from the color changes that less graphene deposits on their shells compared to M-5.

**Figure 5:**
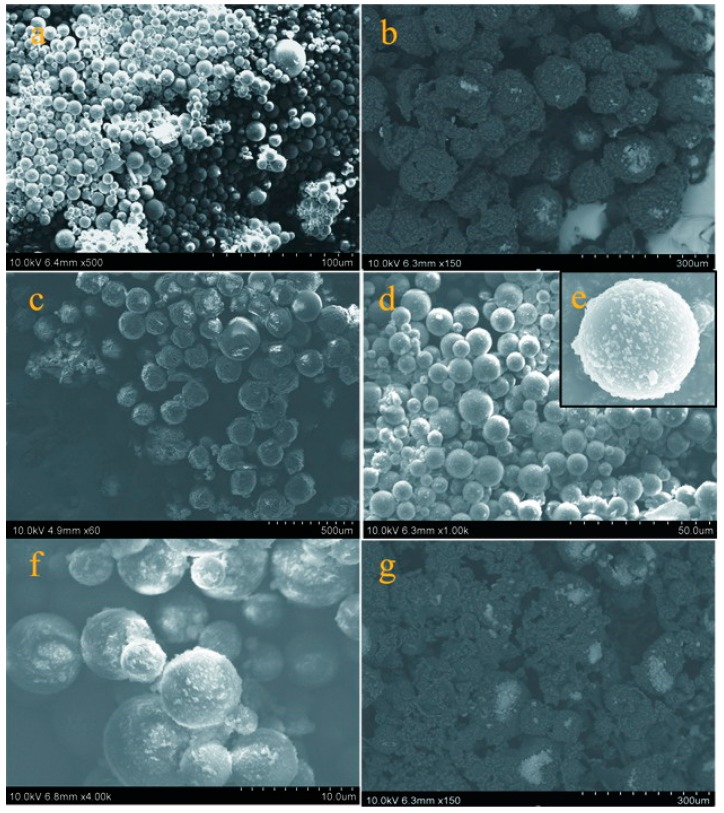
SEM surface morphologies of microcapsules fabricated under various pH values, (**a**) M-0, (**b**) M-3, (**c**) M-4, (**d**,**e**) M-5,(**f**) M-6, and (**g**) M-7.

More microstructure details of shells can be observed from the SEM morphologies of microcapsule samples. Microcapsules (M-0) have a global shape without rupture (Figure 5a), which is consistent with the previous result [9]. The surface morphology of microcapsule samples (M-3 and M-4) can be observed in Figure 5b,c, which have rough surfaces with impurity attachment adheres on the shells. In contrast, the microcapsules (M-5) have a relatively smoother surface and less impurity attachments surround the microcapsules (Figure 5d,e). According to the mechanism of polymerization, the HMMM polymer can not be completely consumed to form the shells. Therefore, the graphene also remains as residue in emulsion entangled with polymer not participating in the reaction during the shell formation process. The surfaces of M-6 and M-7 change to rough again as shown in Figure 5f,g. In particular, the M-7 sample has large amount of impurity between microcapsules. Shell can be considered as a thin membrane protecting the inner core material. These defects and impurities will definitely affect the performance of microcapsules. 

### 3.2. Geometry of the Core-Shell Structure

**Figure 6:**
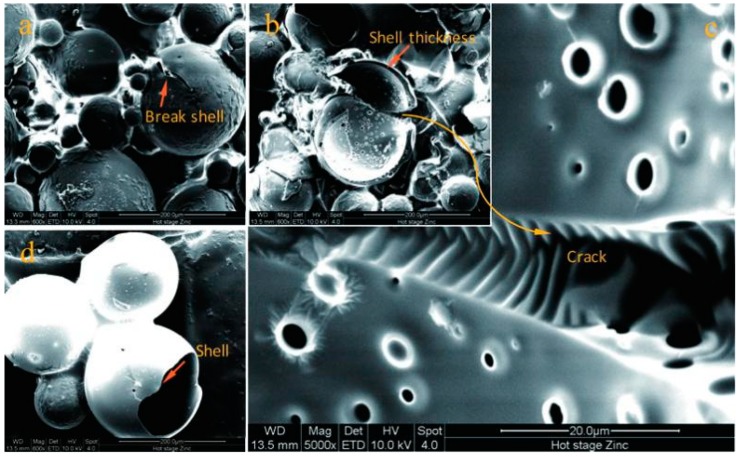
SEM morphologies of typical microcapsules with core-shell structure, (**a**) a microcapsules with a rupture shell, (**b**) a broken shell with measurable thickness, (**c**) the enlarged broken shell, (**d**) the membrane shell of a microcapsule.

Beside the investigation of the surface morphology of microcapsules, it is necessary to prove the core-shell structure of a microcapsule product. At the same time, the geometry of the core-shell structure including the shell thickness and average size should be tested systemically. Both are very important for the physical properties of microcapsules. In this study, the effect of addition of graphene on the geometry also needs to be considered. Figure 6 shows SEM morphology of typical microcapsules with core-shell structure. In Figure 6a, a microcapsule with a crack in the shell is pointed by an arrow. It owns a core-shell structure with a shell thickness of 2 µm (Figure 6b,c). The microstructure of the surface of the microcapsule shell was observed through Figure 6c. The morphology of the shell and core of the microcapsules were observed through an incomplete microcapsule as shown in Figure 6d. The volume of the core of the microcapsule occupies most of the entire microcapsule and the microcapsule has a thinner shell.

**Figure 7:**
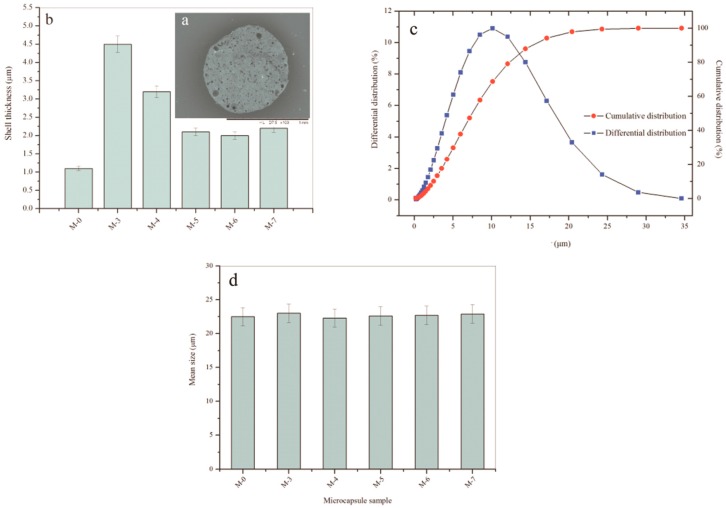
Geometry of the core-shell structure of microcapsule samples, (**a**) SEM morphology of cross-section of microcapsule/gelatin, (**b**) shell thickness of microcapsule samples fabricated under various pH values (M-0, M-3, M-4, M-5, M-6 and M-7), (**c**) cumulative distribution and differential distribution of microcapsule sample (M-0), and (**d**) mean size of microcapsule samples (M-0, M-3, M-4, M-5, M-6 and M-7).

It is hard to measure the shell thickness of a microcapsule because it has a global shape. Another reason is that the microcapsules do not own the same shell thickness fabricated by the hybrid method. In this work, we applied an simple method to successfully measure the shell thickness using an ultramicrotome technology [2]. Figure 7a shows a typical cross-section SEM morphology of microcapsules/gelatin composite. Fifty microcapsules thickness values were measured under a microscope with scale mark and the average data was calculated. Under a stirring speed of 2000 r·min^−1^, the M-0 sample has an average thickness of 1.01 μm as shown in Figure 7b. This value is consistent with the previous result [33]. With the addition of graphene in shell, the shell thickness value of M-3 sharply increases to 4.50 μm. The reason can be attributed to the loss hybrid inorganic/organic microstructure. This phenomenon has further been found in the CaCO_3_/organic shells of microcapsules [33]. With the increasing of pH data of emulsion polymerization, microcapsule samples of M-4, M-5, M-6 and M-7 have thickness values of 3.21, 2.25, 2.20 and 3.32 μm, respectively. All thickness values of these samples have decreased compared to M-3. Under a higher pH value, the polymer has a relatively slower rate of reaction. In other words, the polymer and graphene deposit on core droplets has changed to a low rate. Therefore, the shell may be compactable and smoother. Its conclusion is consistent with the above SEM morphology of microcapsule samples.

Besides the shell thickness, the effect of pH value on the average size of microcapsules also been investigated in this work. It is well known that the microcapsule particles have a normal distribution prepared by an emulsion polymerization method [42]. Figure 7c shows the typical cumulative distribution and differential distribution curves of sample M-0, which was fabricated under a 2000 r·min^−1^ emulsion stirring speed. Nearly all microcapsules have a particle size in the range of 3–20 μm. Usually, the average diameter of microcapsules is determined by the core material emulsion stirring speed and it is not greatly influenced by their shell thickness [2]. The shell is an inorganic/organic composite, and the average size of microcapsules still obeys this rule. The average size of microcapsule samples (M-0, M-3, M-4, M-5, M-6 and M-7) is measured using a laser particle size distribution instrument as shown in Figure 7c. All samples have a average size in a range of 22–23 μm. Both graphene additive and pH values do not greatly affect the average size of microcapsules. This means that the average size of microcapsules is mainly determined by the emulsion speed of core material. 

### 3.3. Chemical Structure of Shells

**Figure 8:**
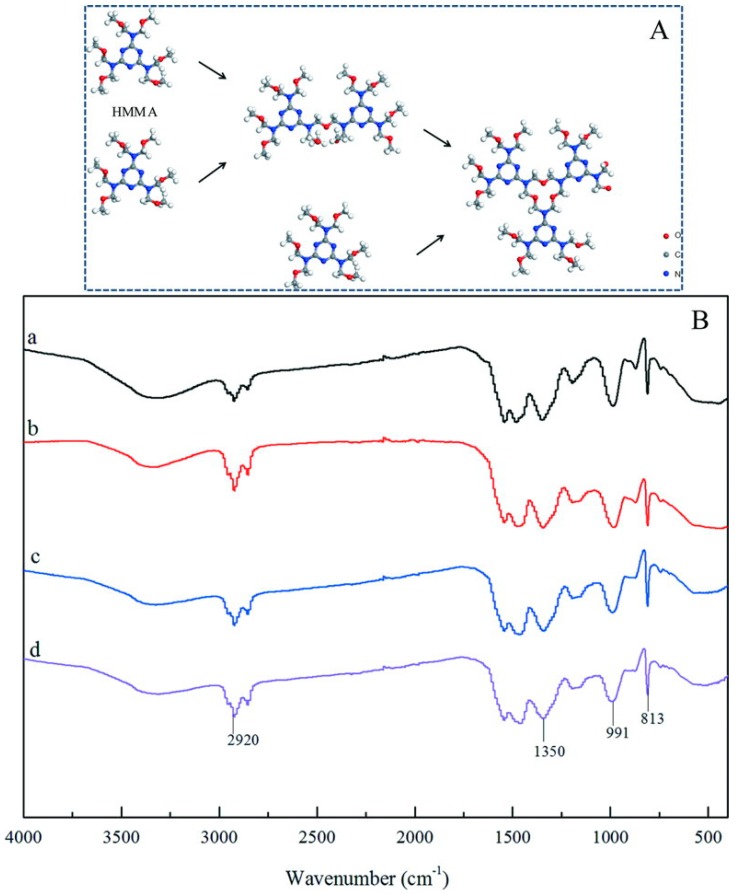
Chemical structures of microcapsule shells fabricated under various pH values, (**A**) molecule structure of HMMM cross-linkage process, (**B**) the FTIR curves of the microcapsule samples prepared under various pH values: (**a**) M-3, (**b**) M-4, (**c**) M-5, and (**d**) M-6.

Figure 8A shows the HMMM molecule polymerization process. The microcapsule shells were formed through hydrolysis of HMMM molecules to remove methyl oxygen (–OCH_3_) under acidic condition. Therefore, the performance of the microcapsule shells is determined by the polymerization of HMMM molecules. The polymerization of HMMM molecules is also determined by the reaction pH value. The FT-IR spectra are used to analyze the chemical structure of microcapsule shells. Figure 8B shows the FT-IR curves of the microcapsule samples prepared under pH = 3 (spectrum a), pH = 4 (spectrum b), pH = 5 (spectrum c) and pH = 6 (spectrum d). The spectra of all samples (a, b, c and d) exhibit four absorption peaks at 2920 cm^−1^, 1350 cm^−1^, 991 cm^−1^, 831 cm^−1^. The absorption peak at 2920 cm^−1^ is attributed to C–H telescopic vibration of methylene. It is difficult to find the absorption peak of the methyl group to prove that the polymerization of HMMM resin is a sufficient chemical reaction. The absorption peaks at 991 cm^−1^ and 1350 cm^−1^ are assigned to the C–N telescopic vibration and C–O–C telescopic vibration in the HMMM resin. No characteristic peak of graphene can be found in the FT-IR curves. The existence of graphene does not affect the characteristic peaks of cross-linked HMMM for all microcapsule samples. The above phenomenon indicates that the pH value does not affect the chemical structure of the microcapsule shell and the graphene is only composited with the HMMM rein by physical cross-linkage.

**Figure 9:**
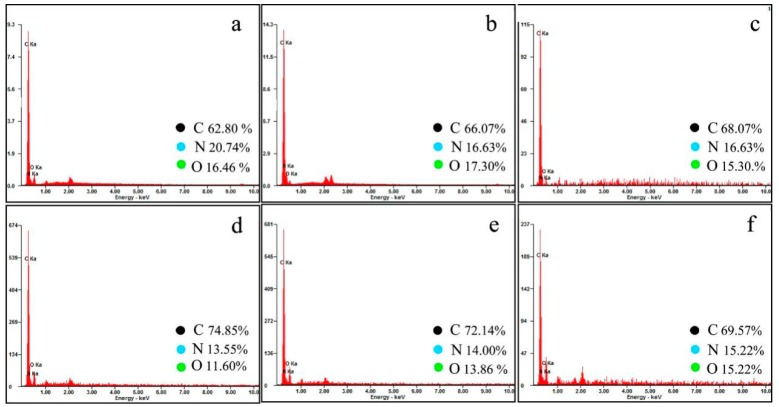
EDS results of microcapsule samples, (**a**) M-0, (**b**) M-3, (**c**) M-4, (**d**) M-5, (**e**) M-6, and (**f**) M-7.

EDS results were applied to evaluate the deposition contents of graphene in shells. Figure 9a show the EDS results of microcapsules without graphene (M-0). The carbon content of the microcapsule shells without adding graphene is 62.80%. Figure 9b–f show the EDS curves of the microcapsules samples prepared under pH = 3 (curve b), pH = 4 (curve c), pH = 5 (curve d), pH = 6 (curve e) and pH = 7 (curve f). The carbon content of these microcapsule shells are 66.07%, 68.07%, 74.85%, 72.14%, and 69.57%. Clearly, M-5 has the largest graphene deposition amount on shells, which are formed under the polymerization condition of pH = 5. 

### 3.4. Microstructure of Graphene in Shells

**Figure 10:**
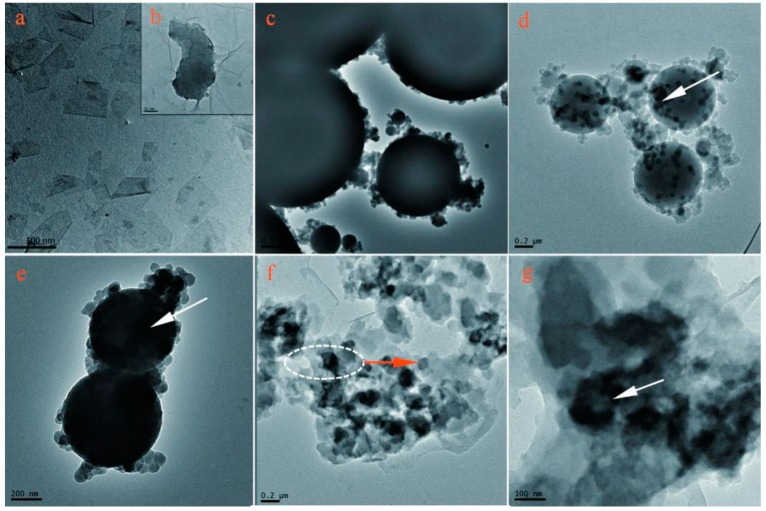
TEM morphologies of microcapsule samples, (**a**,**b**) pure graphene, (**c**) M-0, (**d**) M-4, (**e**) M-5, and (**f**,**g**) shell materials of M-5.

Figure 10 shows the TEM morphologies of graphene on the microcapsule shells. Pure graphene has a single layer structure with size of 100 nm (Figure 10a,b). Microcapsules (M-0) have smooth HMMM shells with compactable global shape (Figure 10c). Comparatively, graphene is found on the shell surface of shells of M-4 and M-5 microcapsule samples as the arrows shown in Figure 10d,e. It can be seen that M-5 possesses more graphene on its surface than M-4. This phenomenon is consistent with the results of EDS. A few microcapsules (M-5) were destroyed and washed with alcohol to remove the core materials obtaining a pure shell structure as shown in Figure 10f,g. The adhesion of graphene and HMMM resin is strong and the graphene is not stripped from HMMM resin. The graphene is assembled and contained in HMMM resin, which is beneficial to improve the electrical and thermal conductivity of microcapsule shells. 

### 3.5. Thermal Properties of Microcapsules

**Figure 11:**
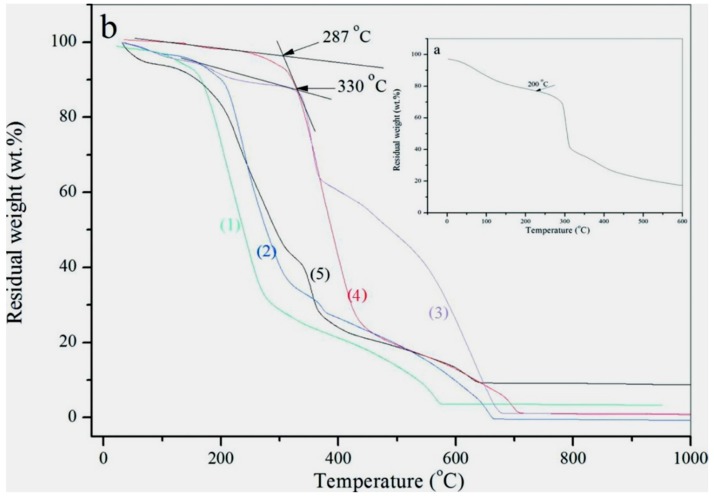
TGA curves of microcapsule samples, (**a**) microcapsules without graphene, (**b**) microcapsule samples prepared under various pH values: (1) M-3, (2) M-4, (3) M-5, (4) M-6 and (5) M-7.

Thermal stability of microcapsules is one of the most important issues in determining the performance of microcapsules. It has been found that the addition of graphene can greatly enhance the thermal stability of the microcapsules. More graphene deposition on shells can lead to a higher thermal stability of microcapsules [2]. The deposition amount of graphene on the microcapsule shells is determined by the pH value during polymerization. The decomposition temperature of microcapsules without addition of graphene (M-0) is 200 °C, as shown in Figure 11a. This result is similar to our reported data in previous work [2]. Figure 11b shows the TGA curves of microcapsule samples prepared under pH = 3 (curve 1), pH = 4 (curve 2), pH = 5 (curve 3), pH = 6 (curve 4) and pH = 7 (curve 5), which have decomposition temperatures of 210 °C, 220 °C, 287 °C, 330 °C, 260 °C, respectively. Obviously, the decomposition temperature of the microcapsule samples with graphene is higher than the microcapsules without graphene (M-0). This phenomenon is similar to the previous research result [9]. M-5 as the highest original decomposition temperature in all samples.

**Figure 12:**
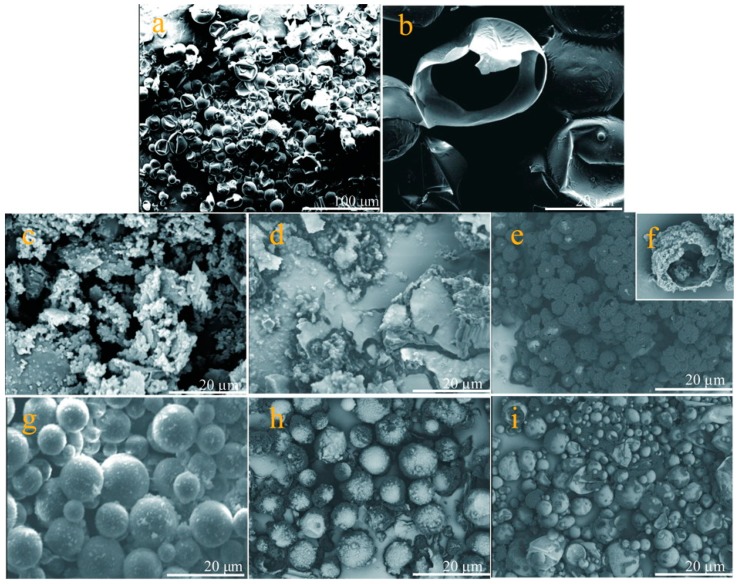
SEM morphologies of microcapsules under 300 °C, (**a**,**b**) M-0, (**c**) M-3, (**d**) M-4, (**e**,**f**) M-5, (**g**,**h**) M-6 and (**i**) M-7.

The morphology of the microcapsules at high temperatures also can reflect the capability of microcapsules resisting heat. Figure 12 shows the SEM morphology of microcapsule samples under a high temperature of 300 °C. Firstly, it can be observed that most microcapsules (M-0) have deformed or broken as shown in Figure 12a. A core-shell structure of broken shell can be recognized directly (Figure 12b). Microcapsules (M-3) have all been completely destroyed as shown in Figure 12c,d. This phenomenon is attributed to their poor compactable shells fabricated under a low pH value. The HMMM may polymerize with a higher rate, leading to too many defects on shells. M-5 sample retains the spherical shape at 300 °C and only a small number of microcapsules were destroyed as shown in Figure 12e. Impurity exists between microcapsules. Figure 12f shows a typical broken microcapsule with a rough surface. M-5 microcapsules do not change global shape, maintaining a smooth surface without break and rupture at 300 °C (Figure 12g). Figure 12h,i shows the SEM morphologies of M-6 and M-7 microcapsule samples suffered an action of 300 °C treatment. Both of them do not own smooth surfaces compared to M-5. Some of microcapsules have broken under such a high temperature. All the above phenomena indicate that M-5 has the best thermal stability, which is prepared under a pH polymerization value of 5.

**Figure 13:**
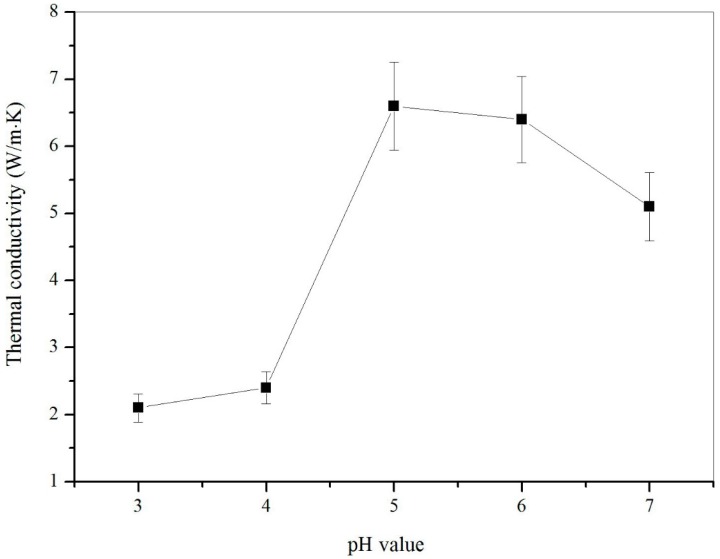
Thermal conductivity of microcapsule samples (M-3, M-4, M-5, M-6 and M-7) fabricated under various pH values.

In previous studies, the greater the deposition of graphene on the shell of the microcapsule samples, the higher the thermal conductivity of the microcapsule samples [9]. Therefore, graphene content in shells can be judged by the thermal conductivity of the microcapsule samples. Figure 13 shows the thermal conductivity of microcapsule samples (M-3, M-4, M-5, M-6, and M-7) fabricated under various pH values. The microcapsule sample with 5% graphene added has a thermal conductivity of up to 6.6805 W·(m·K)^−1^. The significant increase can be discovered from M-4 to M-5. This is due to the increase in the deposition of graphene on the surface of the microcapsule shell. Thermal conductivity starts to increase when the pH values of microcapsules preparation increase from 3 to 5. Thermal conductivity decreases when the pH value of the microcapsule preparation exceeds 5. This phenomenon indicates that the content of graphene on the microcapsule shell is normally distributed with the pH value of the microcapsule preparation. This is consistent with the previous results.

**Figure 14:**
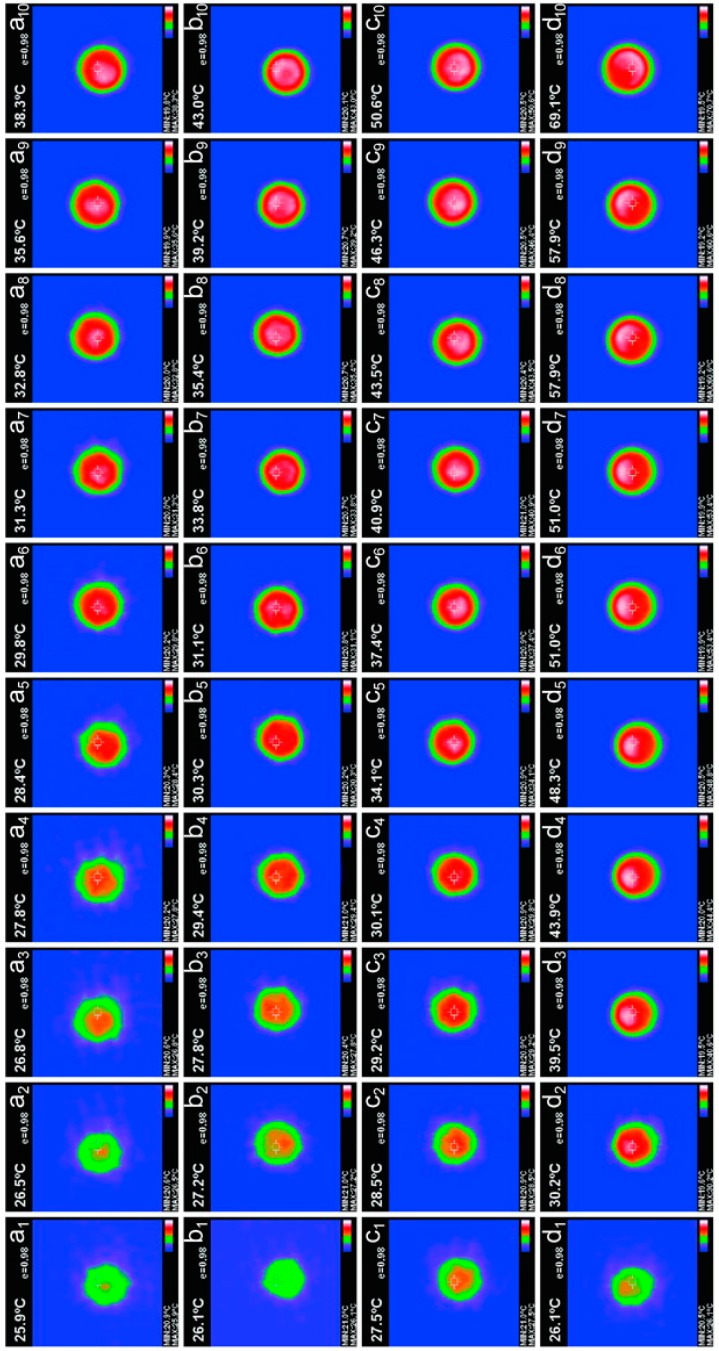
Infrared thermal imaging of microcapsule samples fabricated under various pH values with 5 s intervals in 50 s, (a_1_-a_10_) M-0, (b_1_-b_10_) M-3, (c_1_-c_10_) M-4, and (d_1_-d_10_) M-5.

Another approach was applied to visually observe the thermal conductive character using a constant temperature heating plate with a temperature of 100 °C. A microcapsule sample was put on the heating late for in s, and the thermal images were taken each 5 s. Figure 14 shows the infrared thermal imaging of microcapsule samples of M-0 (a_1_-a_10_), M-3 (b_1_-b_10_), M-4 (c_1_-c_10_), and M-5 (d_1_-d_10_). Sample M-0 has the slowest temperature increasing speed under a constant temperature (100 °C). Its temperature changes from 25.9 °C to 38.3 °C in 50 s. Microcapsules without graphene have a low sensitive to temperature because of poor thermal conductivity of HMMM resin. It can be observed that the thermal conductivity of the microcapsules is improved with the addition of graphene. The temperature of sample M-5 is raised rapidly from 26.1 °C to 69.1 °C in 50 s. This is attributed to the maximum deposition of graphene on the microcapsule shell under the preparation condition of pH = 5. 

### 3.6. Mechanical Property of a Single Microcapsule

Nanoindentation is a robust technique for determination of mechanical properties. By combining the application of low-loads, measuring the resulting displacement, and determining the contact area between the tip of the indenter and the sample a wide range of mechanical properties are able to be measured. The application that drove the innovation of the technique is testing thin film properties for which conventional testing is not feasible. Conventional mechanical testing such as tensile testing or dynamic mechanical analysis (DMA) can only return the average property without any indication of variability across the sample. However, nanoindentation can be utilized for determination of local properties of homogeneous as well as heterogeneous materials. The reduction in sample size requirements has allowed the technique to become broadly applied to products where the manufactured state does not present sufficient material for micro-hardness testing. Applications in this area include medical implants, consumer goods, and packaging. Alternative uses of the technique are used to test devices by utilizing the low-loads and small scale displacements of the nanoindenter [2]. 

Nanoindentation is a robust technique for determination of mechanical properties. By combining the application of low-loads, measuring the resulting displacement, and determining the contact area between the tip of the indenter and the sample, a wide range of mechanical properties are able to be measured. The application that drove the innovation of the technique is testing thin film properties for which conventional testing is not feasible. Conventional mechanical testing such as tensile testing or dynamic mechanical analysis (DMA) can only return the average property without any indication of variability across the sample. However, nanoindentation can be utilized for determination of local homogeneous properties as well as heterogeneous materials. The reduction in sample size requirements has allowed the technique to become broadly applied to products where the manufactured state does not present sufficient material for micro-hardness testing. Applications in this area include medical implants, consumer goods, and packaging. Alternative uses of the technique are used to test devices by utilizing the low-loads and small scale displacements of the nanoindenter [2]. 

**Figure 15:**
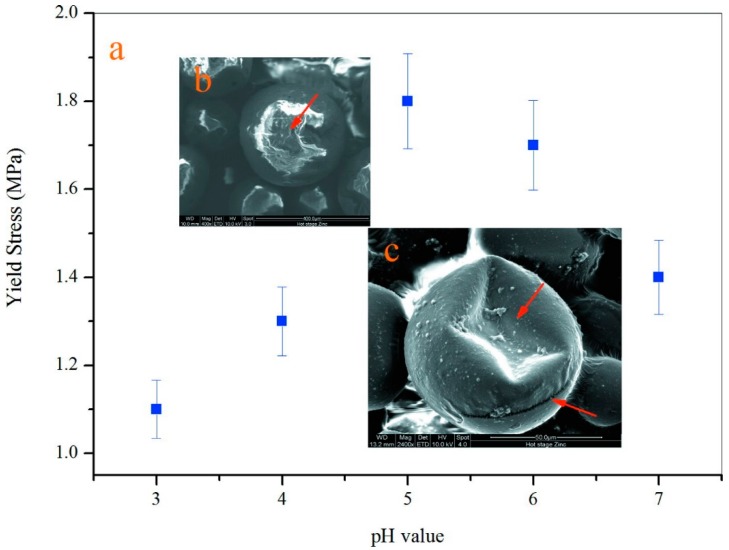
Mechanical property of single microcapsule, (**a**) yield stress values of microcapsule samples tested by nanoindentation, (**b**) SEM morphology of a single microcapsule under pressure, and (**c**) SEM morphology of a single microcapsule with deformation after a yield point.

Nanoindentation technology is now considered as the most convenient and standard method to evaluate the mechanical property of a single microcapsule [2]. The mechanical property is a critical character for a single microcapsule because it greatly influences the stability of microcapsules in application. For example, the shell suffers an inner liquid expansion or the external force. Microcapsules will ultimately rupture under excessive strength and the core material will lose the protection of the shell. It has been found that the mechanical behaviors of shells mainly depend on the thickness and microstructure of the shell. A single microcapsule usually processes an elastic-plastic deformation behavior under pressure. In detail, a microcapsule deforms elastically and then returns to its original shape prior to a yield point when the pressure is unloaded. A permanent deformation appears once the pressure is beyond its yield point. The non-reversible deformation is further expanded until the microcapsule ruptures. Young’s modulus and hardness of the microcapsules can be calculated using nanoindentation technology based on a well-established elastic contact theory [9]. In this work, yield point values of microcapsule samples were measured in order to understand the effect of pH values on the mechanical properties of shells as shown in Figure 15a. The microcapsule has a lowest yield point value of 1.11 MPa fabricated under pH of 3 (M-3). With the increasing of pH values, the yield point value has an increasing trend and the maximum value is about of 1.85 MPa at a pH value of 5. Then yield point values decrease to 1.70 and 1.42 MPa from a pH value of 6 (M-6) to 7 (M-7). This result is consistent with the above microstructure findings that the microcapsules (M-6 and M-7) have more graphene in shells. Figure 15b shows a typical SEM morphology of a single microcapsule without yield after a nanoindentation pressure. The microcapsule still keeps the globe shape with no deformation. Contractually, the microcapsule has an irreversible deformation when the pressure is beyond its yield point, as shown in the SEM morphology of Figure 15c. It indicates that the microcapsule with graphene also follows the elastic-plastic deformation rule. This means that the microcapsule has a clearly elastic deformation beyond a yield point. Obviously, the presence of graphene in the shell dramatically enhances the yield point values of the microcapsule because of the high intrinsic stiffness of layer structure of graphene. Yield point is a mechanical parameter reflecting the resistance capability of plastic deformation. Therefore, graphene addition in shells is a feasible way to enhance the mechanical properties of the microcapsules. Through regulating the pH values of polymerization, the mechanical properties can be controlled due to the various contents of graphene in shells.

### 3.7. Compactability of Shells

The compactability of microcapsules has been widely studied, especially in the controlled release field of medicine. The release behavior greatly depends on the microstructure of shells. It can be regulated through controlling the shell thickness, core/shell ratio, average size, polymerization dynamics and even pore structure. To simplify the complexity of compactability, the microcapsule samples used in this work are considered with the same average diameter because the emulsion stirring rate determined the average diameter of microcapsule particles [42]. Microcapsule samples are prepared with the same core/shell ratio (1/2). Therefore, the effect of polymerization ratio under various pH values is a main factor affecting the release behaviors of paraffin.

**Figure 16:**
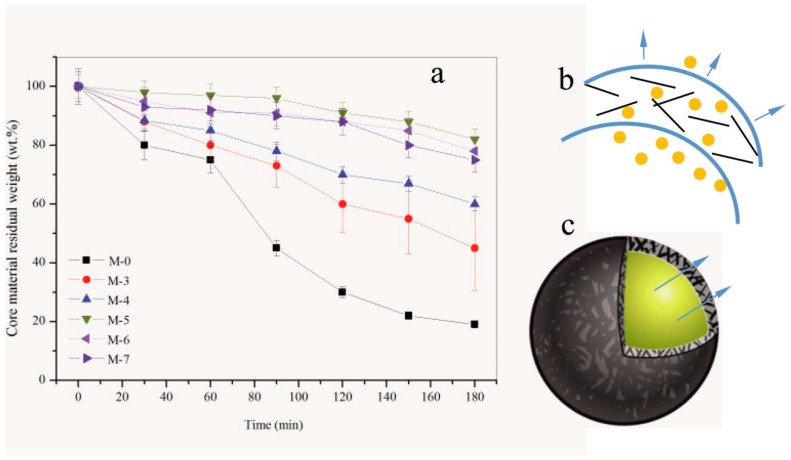
Compactability of shells of microcapsule samples, (**a**) release curves of M-0, M-3, M-4, M-5, M-6, M-7 in alcohol during 180 min, (**b**) the illustration of core material pass through the shell with graphene, and (**c**) the core-shell illustration of a microcapsule.

Figure 16a shows the core material residual weight during 180 of microcapsule samples (M-0, M-3, M-4, M-5, M-6 and M-7) in ethyl alcohol during 180 min. Through comparing the slopes of time-core material residual weight, we know that M-0 has the largest release rate. Its profile has a sharp increase of release after time of 60 min, which implies that majority of the microcapsule may rupture in alcohol on this point. Comparatively, M-5 expresses the best compactability with the lowest release rate. Only 13% core material has been lost before 180 min. This result can be attributed to the reasons illustrated in Figure 16b. One reason is the case that the hybrid graphene/HMMM composite structure has reduced the penetration area of shells. The other reason is that the graphene/HMMM shells have less disfigurement including capillary, micro-cavity or microcrack (Figure 16c). These disfigurements lead the core material to penetrate with a lower resistance. In other words, pH values greatly influence the microstructure and graphene contents of microcapsules. 

## 4. Conclusions

The additive of graphene in polymeric shell of microcapsules is a promising approach to enhance the physicochemical properties of microcapsules because of its well-known superior characteristics. Graphene can be deposits on shells with the combined action of electrostatic adherence and macromolecular chain-entanglement. The self-assembly amount of graphene is greatly influenced by the acid-base equilibrium. The aim of this work was to investigate the hybrid microstructure and properties of self-assembly graphene/HMMM microcapsules containing paraffin affected by the pH value (3–7) in emulation polymerization. The following conclusions can be drawn:

(1) It was proved that several microcapsule samples had been successfully fabricated by the self-assembly process under various pH values in this work. Emulation states and the surface morphologies of microcapsules were observed. The addition amount of graphene was 5% of shell material in the microcapsule. Microcapsules were fabricated under the emulsion speed of 2000 r·min^−1^ with core/shell weight ratio of 2/1.
(2) The addition of graphene did not change the chemical structure of cross-linked HMMM. The existence of graphene had been proved by the EDS results based on the C element in shells. It was found that the microcapsule sample fabricated under pH = 5 owned the largest graphene content in shells. TEM results were used to investigate the state of graphene in a hybrid microstructure.
(3) This microcapsule sample fabricated under pH=5 sample had the best thermal stability and larger thermal conductivity because of this sample had more graphene in shells. Moreover, a single microcapsule of this sample had the largest yield point tested by nanoindentation.
(4) More graphene decreased the penetrability of core material out of shells. Nanoindentation tests proved this sample had the capability of deforming resistance under pressure coming from the composite structure of graphene/polymer structures.

## Figures and Tables

**Table 1:** The microcapsule samples prepared with various conditions.

Samples	Stirring Speed (r·min^−1^)	Core/Shell Ratio	Graphene/Polymer Shell (wt.%)	pH Value
M-0	2000	1/2	0	4
M-3	2000	1/2	5.0%	3
M-4	2000	1/2	5.0%	4
M-5	2000	1/2	5.0%	5
M-6	2000	1/2	5.0%	6
M-7	2000	1/2	5.0%	7
